# Update on the diagnosis and management of COVID-19 in pediatric patients

**DOI:** 10.6061/clinics/2020/e2353

**Published:** 2020-11-25

**Authors:** Ana Paula de Carvalho Panzeri Carlotti, Werther Brunow de Carvalho, Cíntia Johnston, Alfredo Elias Gilio, Heloisa Helena de Sousa Marques, Juliana Ferreira Ferranti, Isadora Souza Rodriguez, Artur Figueiredo Delgado

**Affiliations:** I Departamento de Puericultura e Pediatria, Hospital das Clinicas (HCFMUSP), Faculdade de Medicina de Ribeirao Preto, Universidade de Sao Paulo, Ribeirao Preto, SP, BR; II Departamento de Pediatria, Instituto da Crianca e do Adolescente (ICR), Hospital das Clinicas (HCFMUSP), Faculdade de Medicina, Universidade de Sao Paulo, Sao Paulo, SP, BR; III Departamento de Pediatria, Hospital Universitario, Faculdade de Medicina (FMUSP), Universidade de Sao Paulo, Sao Paulo, SP, BR

**Keywords:** COVID-19, Diagnosis, Respiratory Support, Pharmacological Treatment, Multisystem Inflammatory Syndrome in Children

## Abstract

Coronavirus disease (COVID-19), caused by severe acute respiratory syndrome coronavirus 2 (SARS-CoV-2), became a pandemic in March 2020, affecting millions of people worldwide. However, COVID-19 in pediatric patients represents 1-5% of all cases, and the risk for developing severe disease and critical illness is much lower in children with COVID-19 than in adults. Multisystem inflammatory syndrome in children (MIS-C), a possible complication of COVID-19, has been described as a hyperinflammatory condition with multiorgan involvement similar to that in Kawasaki disease or toxic shock syndrome in children with evidence of SARS-CoV-2 infection. This review presents an update on the diagnostic methods for COVID-19, including reverse-transcriptase polymerase chain reaction (RT-PCR) tests, serology tests, and imaging, and summarizes the current recommendations for the management of the disease. Particular emphasis is placed on respiratory support, which includes noninvasive ventilation and invasive mechanical ventilation strategies according to lung compliance and pattern of lung injury. Pharmacological treatment, including pathogen-targeted drugs and host-directed therapies, has been addressed. The diagnostic criteria and management of MIS-C are also summarized.

## INTRODUCTION

The World Health Organization (WHO) declared the coronavirus disease (COVID-19) a pandemic on March 11, 2020. By August 20, 2020, there were 22,427,939 confirmed cases and 788,030 deaths worldwide. In Brazil, 3,456,652 cases and 111,100 deaths caused by COVID-19 have been reported (1). COVID-19 is infrequent in pediatric patients. Only 2.2% of confirmed COVID-19 cases were reported in patients aged less than 21 years in Brazil (2). In addition, the risk of developing severe disease and critical illness is much lower in children with COVID-19 than in adults (3-7). In European countries, the rates of admission to the pediatric intensive care unit (PICU) were less than 10% (6,7). However, in New York City, 28% of hospitalized pediatric patients with COVID-19 required PICU admission; 84% of them were ≥11 years old. Notably, in this cohort, the most common comorbidities in all hospitalized patients were obesity and asthma (8). Neurological disorders, hypertension, congenital heart disease, diabetes, chromosomal abnormalities, chronic pulmonary disease, chronic kidney disease, malignancies, and immunosuppression have also been described (7-9). A multinational multicenter study from Europe found that the risk factors for PICU admission were age <1 month, male sex, lower respiratory tract infection at presentation, and the presence of pre-existing medical conditions. Although viral co-infection was associated with the requirement of PICU admission on univariable analysis, this association was not significant after multivariable analysis (7). The reported mortality rates for pediatric patients with COVID-19 are remarkably low (< 1%) (7-9). This review presents an update on the diagnostic methods for COVID-19, including reverse-transcriptase polymerase chain reaction (RT-PCR) tests, serology tests, and imaging, and summarizes the current recommendations for the management of the disease.

### Diagnosis

#### Detection of the causative virus of COVID-19

The detection of severe acute respiratory syndrome coronavirus 2 (SARS-CoV-2) RNA from nasopharyngeal swab specimens using real-time RT-PCR is the standard for the diagnosis of COVID-19. The sensitivity of the test is estimated to be 62% on the day of symptom onset and 80.3% three days after symptom onset. Bronchoalveolar lavage fluid specimens provided the highest positive rates (93%), followed by sputum (72%), nasal swabs (63%), and pharyngeal swabs (32%). Viral nucleic acids may also be detected in feces and saliva (10).

#### Serologic methods

The two major antigenic targets for antibodies against SARS-CoV-2 are the spike protein (S) and nucleocapsid protein (N). N Protein is more conserved and can induce antibodies earlier than S protein (11,12). However, neutralizing antibodies are predominantly directed to the S protein (11,12).

Tests used to evaluate immune response may detect either binding or neutralizing antibodies. The tests that detect binding antibodies use purified SARS-CoV-2 proteins with specific reagents to determine IgA, IgM, or IgG. These tests can be of two types: rapid or laboratory tests. Rapid tests can detect IgM, IgG, or total antibodies in plasma, whole blood, or saliva. Laboratory tests use the enzyme-immunoassay or chemiluminescence technique and require specialized personnel and equipment. They can assess IgM, IgG, and IgA separately or in combination, as total antibody. The detection of neutralizing antibodies involves the use of a live virus. These tests can only be performed in laboratories with a high level of biosecurity and hence are not routinely used (11). Currently, virtually all studies on antibody detection have been carried out in adults. Therefore, caution is needed when extrapolating these results to children and adolescents.

Serologic tests can be useful to identify patients who have had COVID-19 or patients with acute infection in the late stage of the disease (between 9 and 14 days after the onset of symptoms). However, these tests can be negative in the initial phase of the disease and their usefulness for diagnosis in the acute phase may be limited (12). However, they can be helpful for testing children and adolescents with multisystem inflammatory syndrome (MIS-C). In fact, it has been shown that children with MIS-C have significantly higher titers of SARS-CoV-2 binding and neutralizing antibodies than do children with COVID-19 only. In addition, antibody titers correlated with systemic inflammation and clinical outcomes (13). Serologic tests can also be beneficial for epidemiological inquiries because they assist in determining whether the individual has been previously infected, regardless of past history of symptoms suggestive of COVID-19 (11).

The usefulness of serologic tests depends on the sensitivity, specificity, and prevalence of the disease in the population. The positive predictive value, which is the probability that individuals with a positive test result are truly positive, varies according to the prevalence of the disease in the population (pre-test probability). Thus, even tests with high specificity when used in places with a low prevalence of the disease (5% to 25%) have a high probability of false positives. Therefore, serological tests should not be used as the only test to diagnose or exclude active infection by SARS-CoV-2 (11).

Most COVID-19 patients show seroconversion two weeks after symptom onset, and a majority have detectable antibodies after 28 days (12). The peaks of IgA and IgM occur 7 to 14 days after the onset of symptoms, whereas the peak of IgG occurs simultaneously with the peak of IgA and IgM in some cases or a little later, reaching a plateau between 15 and 21 days (14). A systematic review found that IgM appears in 23%, 58%, and 75% of cases after 1, 2, and 3 weeks, respectively, while IgG appears in 30%, 66%, and 88% of cases after 1, 2, and 3 weeks, respectively (15). Little is known about IgA response. IgM detection without the detection of IgG is uncommon (11). IgM is also associated with false-positive results (12). Some patients have weaker seroconversion. The severity of the disease may play an important role in antibody response. Critical patients usually have a later response, but they are more robust (16). The duration of detectable antibodies is uncertain (11).

Many tests that are currently available have obtained emergency authorization for their use, without external validation. These are mostly qualitative or semi-quantitative tests. Virtually all of them have only undergone validation by the manufacturer, often with a small number of samples (12). In Brazil, the Health Surveillance Agency has approved 11 serological tests for identifying antibodies to the SARS-CoV-2 N protein. Of the 11 tests, 9 are rapid tests. Among the tests that detect IgM, the sensitivity varies from 76% to 87% and the specificity ranges from 96% to 98%. Tests that measure IgG have a sensitivity that varies from 90% to 99% and the specificity from 97% to 99% (17).

Presently, serological tests should be interpreted with great caution because there are many gaps in knowledge. We still do not know exactly the correlation between the results of serology and protection against the disease and the degree to which these tests can cross-react with antibodies to other coronaviruses, especially for tests using the N protein (12).

#### Imaging findings

Chest radiography findings may be normal in the early phase of the illness or in patients with mild disease. Nevertheless, radiographic abnormalities are frequent during hospitalization with more alterations within 10-12 days after onset of symptoms. Airspace opacities are frequent, and chest radiography can show signs of pneumonia, including interstitial alterations, usually affecting peripheral areas. Ground-glass opacities and consolidation may be observed in severe cases. Lesions are most often bilateral, peripheral, and lower-zone predominant and pleural effusion is uncommon (3%) (18-22).

Lung ultrasonography is very useful in patients with pneumonia for diagnosis, monitoring, and follow-up. Wireless equipment and tablets can be more adequate apparatus for use in COVID-19 cases. Advanced COVID-19 pneumonia is characterized by evident consolidation, particularly in the posterobasal regions. Possible findings include multiple B-lines (ranging from focal to diffuse with spared areas, representing thickened subpleural interlobular septa); irregular, thickened pleural line with scattered discontinuities; and subpleural consolidations and alveolar consolidation, with restitution of aeration during recovery (reappearance of bilateral A-lines) (22,23).

Early chest computed tomography (CT) examination is very important in symptomatic patients. Some pediatric patients with a negative test for SARS-CoV-2 nucleic acid are managed as suspected COVID-19 cases according to the typical lesions shown on chest CT, which could provide evidence not only for early treatment but also for effectively isolating the source of infection. Typical manifestations include unilateral or bilateral subpleural ground-glass opacities and consolidations with surrounding halo signs. As consolidations with surrounding halo signs account for up to 50% of cases, they should be considered as typical signs in pediatric patients. In children, there is less diversity in CT scan findings compared to that in adults, with only a small number of pediatric patients having multiple lobes affected. Chest CT can also exhibit ground-glass opacities and segmental consolidation in both lungs. Children presenting with severe infection may show bilateral lobar consolidation (2). CT imaging of COVID-19 infection should be differentiated from other viral pneumonia caused by influenza virus, parainfluenza virus, respiratory syncytial virus, and adenovirus (20-22,24).

Electrical impedance tomography (EIT) is a bedside monitoring tool that noninvasively visualizes local ventilation and, arguably, lung perfusion distribution. It is a noninvasive radiation-free monitoring technique that provides images based on the tissue electrical conductivity of the chest (25). Evidence suggests that EIT may be useful for positive end-expiratory pressure (PEEP) titration in patients with acute respiratory distress syndrome (ARDS) related to COVID-19 (26).

#### Diagnostic criteria for Multisystem Inflammatory Syndrome in children (MIS-C)

In early May 2020, a hyperinflammatory syndrome with multiorgan involvement similar to that in Kawasaki disease, Kawasaki disease shock syndrome, or toxic shock syndrome was reported in children with evidence of SARS-CoV-2 infection. The diagnosis of MIS-C, based on the case definition proposed by the United States Centers for Disease Control and Prevention (CDC) should include (27,28) as follows:

- Patients aged <21 years with fever (>38.0°C for ≥24 h or report of subjective fever lasting ≥24 h) with evidence of serious illness requiring hospitalization, with multisystem involvement of more than two organs (cardiac, kidney, respiratory, hematologic, gastrointestinal, dermatologic, or neurological) and laboratory evidence of inflammation by alteration of one or more of the following markers: increased levels of C-reactive protein, erythrocyte sedimentation rate, procalcitonin, fibrinogen, D-dimer, ferritin, lactic dehydrogenase, or interleukin-6 (IL-6); increased number of neutrophils; reduced number of lymphocytes; and low albumin levels

AND

- No other plausible diagnosis

AND

- Current or recent diagnosis of SARS-CoV-2 infection by RT-PCR, serology, or antigen test or exposure to a suspected or confirmed case of COVID-19 within four weeks of symptom onset.

Other conditions should also be considered as MIS-C, such as patients with complete or incomplete Kawasaki disease, but who meet the criteria for the case definition as well as any pediatric death with evidence of infection caused by SARS-CoV-2.

The diagnostic criteria of MIS-C according to the Royal College of Pediatrics and Child Health (United Kingdom) include persistent fever (>38.5°C), signs of inflammation (neutrophilia, elevated C-reactive protein, lymphopenia), and evidence of single or multiorgan dysfunction (cardiovascular, respiratory, kidney, gastrointestinal, or neurological). Children fulfilling full or partial criteria for Kawasaki disease may also be included. Any other microbial causes should be excluded (bacterial sepsis, staphylococcal or streptococcal shock syndromes, or enterovirus myocarditis). The SARS-CoV-2 PCR test results may be positive or negative (28).

The WHO criteria for MIS-C case definition are:

- Patients 0-19 years old with fever for >3 days AND two of the following:
-
  - Rash or bilateral nonpurulent conjunctivitis or signs of mucocutaneous inflammation (oral, hands, or feet)
  - Hypotension or shock
  - Myocardial dysfunction, pericarditis, valvulitis, or coronary abnormalities (including echocardiogram findings or elevated troponin/N-terminal pro-B type natriuretic peptide concentrations)
  - Coagulopathy (elevated prothrombin time, activated partial thromboplastin time, D-dimers)
  - Diarrhea, vomiting, or abdominal pain

AND

- Raised markers of inflammation (erythrocyte sedimentation rate, C-reactive protein, or procalcitonin)

AND

- No other microbial cause (bacterial sepsis, staphylococcal, or streptococcal shock syndromes)

AND

- Evidence of COVID-19 (RT-PCR test, antigen test, or serology) or likely contact with patients with COVID-19

Consider MIS-C in children with features of typical or atypical Kawasaki disease or toxic shock syndrome (28).

Since the beginning of August 2020, the Ministry of Health of Brazil has implemented the compulsory notification requirement for MIS-C cases.

### Treatment

#### Respiratory support

Noninvasive ventilation (NIV) and high-flow nasal oxygen therapy (HFNO)

At the beginning of the COVID-19 pandemic, the use of NIV as well as HFNO was contraindicated or restricted because of little scientific knowledge about COVID-19 (29). In addition, there was a need to establish processes that could guarantee the biosafety of healthcare professionals involved in insertion, monitoring, and removal of the respiratory support devices, which determine the risk of aerosolization and viral dispersion in the environment and, consequently, the contamination of the multidisciplinary team (30).

However, according to the evolution of professional biosafety care, HFNO has been recommended for COVID-19 patients with mild acute respiratory failure, whereas NIV should be used in patients with moderate acute respiratory failure, provided adequate precautions are taken: a) Level 3 personal protective equipment is available for the healthcare team; b) use of double-circuit (inspiratory and expiratory limbs) NIV devices; c) use of full face, oronasal, or helmet interfaces; d) use of heat and moisture exchanger filter near the patient interface (in the Y) or high-efficiency particulate arrestance filter in the exhalation limb of the circuit; d) the patient is in an isolation room with negative pressure or a cohort unit in a well-ventilated room (31). In addition, it is suggested to keep the patient at an exhaled air dispersion distance, as described in Tables 1 and 2 (32).

Studies on the applicability of NIV in pediatric patients with COVID-19 are not yet available in the literature. However, based on clinical experience, a 30-90-minute trial has been recommended to determine the continuity of this type of ventilatory support or the requirement for tracheal intubation (33). It has been suggested to start with low settings in bi-level mode (inspiratory positive airway pressure [IPAP] 8—12 cmH2O/expiratory positive airway pressure [EPAP] 4—6 cmH2O), with a gradual increase in pressure (IPAP by 2 cmH2O; EPAP by 1 cmH2O) to maintain pulse oxygen saturation (SpO2) ⩾92%, and continuously evaluating patients’ respiratory function and oxygen saturation. HFNO may reduce the need for invasive ventilation and escalation of therapy compared with tracheal intubation in COVID-19 patients with acute hypoxemic respiratory failure. However, these benefits must be balanced against the unknown risk of airborne transmission (34).

### Invasive respiratory support

**Figure 1:**
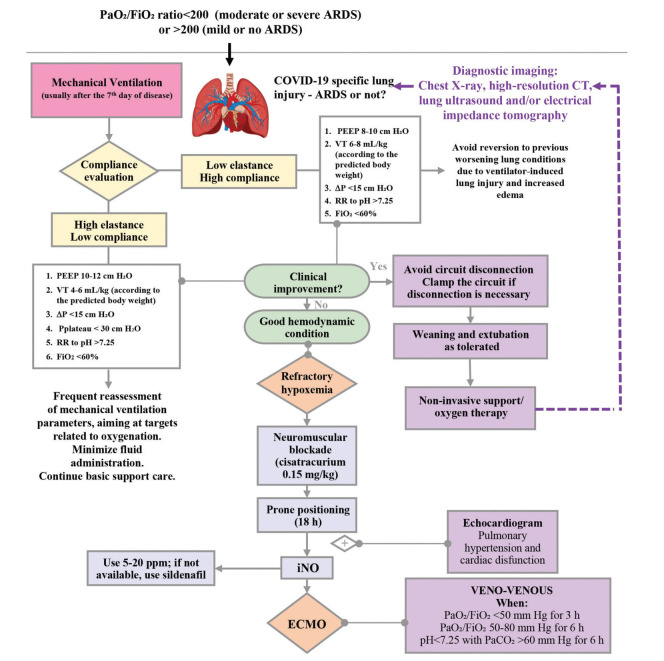
Flow diagram of invasive mechanical ventilation for COVID-19 patients.

Mechanical ventilation is an essential tool for the treatment of critically ill patients with COVID-19. Indeed, it seems that the hypoxemic respiratory failure resulting from COVID-19 may be different from the usual types of ARDS (35). Two different categories of lung injury have been described; thus, different ventilation strategies are required according to the pattern of lung injury. We recommend a compliance evaluation at least twice a day using EIT without perfusion analysis if available or performing respiratory mechanics to determine which type of lung injury seems predominant. For high compliance, we suggest using PEEP around 8 cm H_2_O for infants, toddlers, and preschoolers, and PEEP around 10 cm H_2_O for children older than 6 years. The tidal volume should be approximately 6-8 mL/kg of predicted body weight (PBW) (36,37) and up to 8-9 mL/kg PBW may be tolerated. Inspiratory time should be age-appropriate with a decelerating flow. The plateau pressure should be as low as possible, maintaining adequate PEEP and maximum driving pressure of 15 cm H_2_O. The respiratory rate should be sufficient to maintain a pH >7.25, and inspired oxygen fraction (FiO_2_) should be <60%. We do not recommend using recruitment maneuvers for these types of patients. Patients with low compliance should be managed using protective ventilation (35) (Figure 1). We suggest using pressure-controlled ventilation with a tidal volume of 4-6 mL/kg PBW, initial PEEP around 10 cm H_2_O and titrated up to 12 cm H_2_O (similar to the low PEEP table from ARDS network) (38), keeping in mind that excessive amounts of fluids may influence adequate PEEP titration. The plateau pressure should be <30 cm H_2_O, FiO_2_ <60% and the inspiratory time should be age-appropriate with a decelerating flow and respiratory rate sufficient to maintain a pH >7.25. We recommend using permissive hypercapnia (pH >7.20), with monitoring of PCO_2_ by capnography (preferably volumetric capnography). Goal SpO_2_ is 93-96%, as supraphysiologic arterial oxygen saturation may be associated with higher mortality (39).

Some patients may present with wheezing or clinical features of lower airway obstruction, and they should be treated with bronchodilators (preferably dosimetric or spray inhalers) and magnesium sulfate (initial dosing of 50 mg/kg, as an infusion for over at least 30 min), as needed. For these patients, we recommend using low respiratory rates, inspiratory/expiratory time ratio of 1:3-1:4, and the lowest PEEP possible to avoid auto-PEEP.

#### Refractory hypoxemia

A patient can be considered to have refractory hypoxemia when SpO2 is <90%, with FiO_2_ >60%, driving pressure >15 cm H2O, plateau >30 cm H2O, and PCO2 does not respond to ventilation or dead space reduction. For these patients, we recommend using the following strategies:

- Prone position: It is known that the prone position decreases the ventilation/perfusion (V/Q) mismatch due to pulmonary shunting (35) and optimizes ventilation. We suggest a trial of prone positioning and, if there is a good response (PaO_2_ increases more than 20 mmHg, SpO_2_ >93%, improvement in EIT and in ventilation), it should be maintained for 18 h. If the team’s workload is high, the patient can be kept in the prone position for up to 24 h. If the prone position is not possible, the patient should be lateralized, keeping the more affected lung upwards.
- Nitric oxide: Inhaled nitric oxide (iNO) is a potent pulmonary vasodilator that has been used as rescue therapy in patients with refractory hypoxemia because it enhances pulmonary perfusion in areas with better ventilation; likewise, it can be used in patients with SARS-CoV-2 pulmonary thrombosis. A transient improvement in the PaO_2_/FiO_2_ ratio may occur. We suggest an initial dosing of 5 parts per million (ppm), up to 20 ppm. If nitric oxide is not available, we suggest using sildenafil (0.5-2 mg/kg/dose every 4-6 h, with a maximum of 20 mg/dose every 8 hours).
- Recruitment maneuvers: We do not recommend the routine use of recruitment maneuvers. They should only be performed with adequate monitoring, team expertise, and good clinical condition. We suggest a trial of step-by-step titration of PEEP. Beware of mechanical complications, such as barotrauma and hemodynamic instability.
- High-frequency oscillatory ventilation: We recommend against its use because of the risk of contamination of healthcare workers and other caregivers. It may be used in units with adequate isolation.
- Extracorporeal membrane oxygenation (ECMO): Consider veno-venous ECMO for patients hospitalized in experienced centers, when PaO_2_/FiO_2_ <50 mmHg for 3 h, or PaO_2_/FiO_2_ 50-80 mmHg for 6 h, and pH <7.25 with PaCO_2_ >60 mmHg for 6 h, in the absence of relative contraindications (multiple organ dysfunction, severe neurologic dysfunction, or palliative care) (40).

#### Weaning/extubation

Similar to intubation, extubation is a critical step in the care of critically ill patients with COVID-19. We recommend achieving very low ventilator settings before extubation (FiO_2_ <30%, pressure support ≤10 cm H2O, and PEEP ≤6 cm H2O). We also suggest performing a spontaneous breathing trial for better evaluation of extubation success. It is also important to test gag reflex and other airway protection reflexes before extubation, as there have been reports of re-intubation in patients with aspiration or excessive airway secretions, some of which require bronchoscopy (41).

#### Pharmacological Treatment

The pharmacological treatment of COVID-19 includes pathogen-targeted drugs (antivirals, convalescent plasma) and host-directed therapies (anti-inflammatory and immunomodulatory agents) (42).

Remdesivir has been recommended for the treatment of COVID-19 in hospitalized patients with SpO_2_≤94% in room air or in patients who require supplemental oxygen (43). A multinational randomized, placebo-controlled trial of hospitalized patients with COVID-19 showed that patients with severe disease who received remdesivir had a shorter time to clinical recovery than did those who received placebo (44). However, a randomized double-blind placebo-controlled trial from China showed no significant difference in time to clinical improvement, 28-day mortality, or time to viral clearance in adults admitted to hospitals with severe COVID-19 who were treated with remdesivir compared with placebo (45). Several clinical trials are underway to evaluate the effectiveness and safety of remdesivir in patients with COVID-19, including one trial in pediatric patients from 0 to 18 years (43,46). Currently, there are insufficient data to recommend the use of remdesivir in patients with mild or moderate COVID-19 (43).

Lopinavir/ritonavir, a combination agent approved for treating HIV, has *in vitro* activity against SARS-CoV 3-chymotrypsin-like protease, which appears to be highly conserved in SARS-CoV-2. However, because of its pharmacodynamics, higher than tolerable levels of the drug might be required to achieve *in vivo* inhibition (43,46). An open-label randomized controlled trial of lopinavir/ritonavir compared with the standard of care in COVID-19 did not show significant differences in time to clinical improvement, viral clearance, or 28-day mortality between groups (47). Currently, lopinavir/ritonavir is not recommended for the treatment of COVID-19 (43).

Chloroquine or hydroxychloroquine are not recommended for the treatment of COVID-19 outside of a hospital because of the risk of cardiac arrhythmia and they are not recommended in children (43). A multicenter retrospective observational study in adults showed that hydroxychloroquine and hydroxychloroquine+azithromycin yielded a 66% and 71% reduction in mortality hazard ratio, respectively, compared with neither treatment being used (48). However, a large multicenter randomized controlled trial in adults showed that the use of hydroxychloroquine for the treatment of hospitalized patients with COVID-19 was not associated with a reduction in the 28-day mortality compared with the standards of care for such patients (49).

Azithromycin has been used for the treatment of COVID-19 in combination with hydroxychloroquine. A clinical trial is currently evaluating azithromycin as a monotherapy (43). Currently, azithromycin is not recommended for the treatment of COVID-19.

To date, data are insufficient to recommend for or against the use of convalescent plasma for the treatment of COVID-19. Clinical trials for the use of convalescent plasma in COVID-19 in both adults and children are ongoing (43).

Dexamethasone has been recommended for the treatment of COVID-19 in patients who require supplemental oxygen or those who are mechanically ventilated. The use of dexamethasone is not recommended for patients with COVID-19 who do not require supplemental oxygen (43). A randomized controlled open-label trial showed that dexamethasone reduced the 28-day mortality in patients hospitalized with COVID-19 receiving invasive mechanical ventilation or oxygen, but not among patients not receiving respiratory support (50). However, these results should be interpreted with caution for patients less than 18 years old, as the study did not include a significant number of pediatric patients. Therefore, the use of dexamethasone may be beneficial for pediatric patients with COVID-19 who are on mechanical ventilation, but it is usually not recommended for pediatric patients who require low levels of oxygen support (*i.e.*, nasal cannula only) (43).

Tocilizumab is a monoclonal antibody against the IL-6 receptor. There are insufficient data to recommend for or against the use of IL-6 inhibitors in patients with COVID-19 (43). A prospective open-label multicenter study from Italy found that the use of tocilizumab in hospitalized adults with severe COVID-19 was associated with a reduction in ferritin, C-reactive protein, and D-dimer concentrations. Additionally, an increased likelihood of survival was observed when tocilizumab was administered within 6 days of hospitalization (51).

The use of non-SARS-CoV-2 specific intravenous (IV) immunoglobulin is not recommended for the treatment of COVID-19. However, it has been used for the treatment of MIS-C related to COVID-19, especially in patients with Kawasaki disease. No clinical data are available on the use of specific SARS-CoV-2 immunoglobulin for the treatment of COVID-19 (43).

Venous thromboembolism prophylaxis should be prescribed for pediatric patients with COVID-19 according to the recommendations for those who have been hospitalized for other indications unless contraindicated (active bleeding or platelet count <50,000/mm^3^) (43). Enoxaparin 0.5-1 mg/kg every 12 h subcutaneously can be administered for thromboembolism prophylaxis; patients with hemodynamic instability or severe renal dysfunction should receive a continuous intravenous infusion of unfractionated heparin, starting at 10 IU/kg/h (52).

Table 3 summarizes the pharmacological treatment for COVID-19.

#### Treatment of MIS-C

Treatment of MIS-C consists of supportive care and the use of anti-inflammatory agents. Supportive care includes fluid resuscitation, inotropic support, and respiratory support; in rare cases, extracorporeal membrane oxygenation may be needed. Broad-spectrum antibiotics should be used to treat potential sepsis while awaiting culture results. Management of the underlying inflammatory process involves the use of IV immunoglobulin (1-2 g/kg) and corticosteroids (methylprednisolone 1-2 mg/kg/day IV or pulse 30 mg/kg IV for patients with shock requiring high doses or multiple inotropes and/or vasopressors). Anakinra (IL-1 receptor antagonist) 5 mg/kg/day IV or subcutaneously may be considered for the treatment of MIS-C refractory to IV immunoglobulin and corticosteroids. Alternatively, tocilizumab (12 mg/kg IV for patients <30 kg, 8 mg/kg IV for patients ≥30 kg; max 800 mg) can also be used in children with signs of hyperinflammation. Aspirin (30-50 mg/kg/day until afebrile for 48 hours, then 3-5 mg/kg/day) should be used in patients with MIS-C and Kawasaki-like disease and/or thrombocytosis (platelet count≥450,000/mm^3^). Patients with coronary artery aneurysms and a maximal z-score of 2.5-10 should be treated with low-dose aspirin. Patients with a z-score ≥10 should be treated with low-dose aspirin and therapeutic anticoagulation with enoxaparin or warfarin. Patients with MIS-C and documented thrombosis or an ejection fraction <35% should receive therapeutic anticoagulation with enoxaparin until at least 2 weeks after discharge from the hospital (28,53).

## AUTHOR CONTRIBUTIONS

Carlotti APCP, Carvalho WB, Johnston C, Gilio AE, Marques HHS, Ferranti JF, Rodriguez IS and Delgado AF were responsible for study conceptualization, supervision, original manuscript drafting, editing, and review.

## Figures and Tables

**Table 1:** Maximum exhaled air dispersion distance using different methods of oxygen administration.

Oxygen therapy	Maximum exhaled air dispersion distance
Nasal catheter (5 L/min)	100 cm
Oronasal mask (4 L/min)	40 cm
Venturi mask (40% FiO_2_)	33 cm
Non-rebreathing mask (12 L/min)	10 cm
HFNO (60 L/min)	17 to 62 cm

Adapted from Ferioli M et al., 2020 (32).

**Table 2:** Maximum exhaled air dispersion distance using different noninvasive ventilatory support strategies.

Noninvasive ventilation	Maximum exhaled air dispersion distance
CPAP via oronasal mask (20 cmH_2_O)	Insignificant dispersion
CPAP via nasal prong	33 cm
NIV via full face mask(IPAP 18 cmH_2_O/EPAP 5 cmH_2_O)	92 cm
NIV via helmet without tight air cushion (IPAP 20 cmH_2_O/EPAP 10 cmH_2_O)	27 cm
NIV via helmet with tight air cushion (IPAP 20 cmH_2_O/EPAP 10 cmH_2_O)	Insignificant dispersion

Adapted from Ferioli M et al., 2020 (32).

**Table 3:** Pharmacological treatment for COVID-19.

Agent	Mechanism of action	Dose	Adverse effects
Remdesivir	Inhibition of viral RNA polymerase	Adult and pediatric patients >40 kg NOT on invasive mechanical ventilation: 200 mg IV on Day 1 followed by 100 mg IV on Days 2-5 Pediatric patients <40 kg NOT on invasive mechanical ventilation: 5 mg/kg IV on Day 1 followed by 2.5 mg/kg IV on Days 2-5 Patients ON mechanical ventilation and/or ECMO and patients with no clinical improvement after 5 days of treatment: extend the duration of therapy to 10 days Administration: IV infusion over 30-120 minutes	Transient elevations in transaminases, acute kidney injury, gastrointestinal symptoms (nausea, vomiting) Not recommended for patients with GFR <30 mL/min/1.73 m^2^ or patients on dialysis or those with increased plasma concentrations of alanine aminotransferase or aspartate aminotransferase (>5 times the upper limit of normal)
Lopinavir/ritonavir	Inhibition of 3-chymotrypsin-like protease	Lopinavir 300 mg/m^2^ (max. 400 mg) plus ritonavir 75 mg/m^2^ (max. 100 mg) PO twice daily for up to 14 days	Gastrointestinal effects (nausea, vomiting, diarrhea), transaminase elevation, prolongation of QTc interval, torsades de pointes, PR interval prolongation
Chloroquine phosphate/hydroxychloroquine sulfate	Blocks viral entry by inhibition of glycosylation of the cellular angiotensin-converting enzyme 2 receptor, proteolytic processing, and endosomal acidification. Immunomodulatory effects: inhibits the production of cytokines, autophagy, and lysosome activity	Adults and adolescents ≥50 kg:500 mg of chloroquine phosphate PO every 12-24 h for 5-10 days (500 mg of chloroquine phosphate =300 mg of chloroquine base) 400 mg of hydroxychloroquine sulfate PO every 12 h on Day 1, then 200 mg PO every 12 h for 4 days (200 mg of hydroxychloroquine sulfate=155 mg hydroxychloroquine base) Children: 5-10 mg/kg/day of chloroquine base for 5-10 days	Abdominal cramps, diarrhea, nausea, vomiting, prolongation of QTc interval (additive effect with azithromycin and fluoroquinolones), hemolysis (especially in patients with G6PD deficiency), hypoglycemia, retinal toxicity, neuropsychiatric and central nervous system effects, idiosyncratic reactions
Azithromycin	Reduction of viral replication by induction of interferon-stimulated genes. Stimulation of neutrophil activation and attenuation of inflammatory cytokines in epithelial cells and airway smooth muscle cells	10 mg/kg (max. 500 mg) PO on Day 1 followed by 5 mg/kg (max. 250 mg) PO on Days 2-5	Gastrointestinal symptoms (diarrhea, nausea, vomiting), hepatotoxicity, prolongation of the QTc interval (additive effect with chloroquine/hydroxychloroquine)
Convalescent plasma	Plasma containing antibodies to SARS-CoV-2 may help suppress the virus and modulate the inflammatory response	Adults: Transfusion of 200-500 mL of convalescent plasma (ABO-compatible, preferentially)	Transfusion-associated circulatory overload, transfusion-related acute lung injury, allergic reactions, transmission of infectious pathogens, and red cell alloimmunization
Dexamethasone	Anti-inflammatory effects, suppression of cytokine-related lung injury	Adults: 6 mg/day IV or PO for up to 10 days	Hyperglycemia, hypertension, secondary infections, psychiatric disorders, adrenal insufficiency, myopathy (particularly if used with neuromuscular blockers)
Tocilizumab	IL-6 inhibition, reduction of cytokine storm	400 mg or 8 mg/kg IV, 1-2 doses; second dose 8-12 h after the first dose, if inadequate response.Administration: IV infusion over 60 minutes	Increased aspartate aminotransferase, neutropenia, thrombocytopenia, risk for serious infections (including tuberculosis), hypertension, hypersensitivity reactions
